# Which Fish Should I Eat? Perspectives Influencing Fish Consumption Choices

**DOI:** 10.1289/ehp.1104500

**Published:** 2012-02-22

**Authors:** Emily Oken, Anna L. Choi, Margaret R. Karagas, Koenraad Mariën, Christoph M. Rheinberger, Rita Schoeny, Elsie Sunderland, Susan Korrick

**Affiliations:** 1 Department of Population Medicine, Harvard Pilgrim Health Care Institute and Harvard Medical School, Boston, Massachusetts, USA; 2 Department of Environmental Health, Harvard School of Public Health, Boston, Massachusetts, USA; 3 Section of Biostatistics and Epidemiology, Department of Community and Family Medicine, Dartmouth Medical School, Lebanon, New Hampshire, USA; 4 Washington State Department of Health, Olympia, Washington, USA; 5 Laboratoire d’economie des ressources naturelles, Institut national de la recherche agronomique, Toulouse School of Economics, Toulouse, France; 6 U.S. Environmental Protection Agency, Washington, DC, USA; 7 Channing Laboratory, Department of Medicine, Brigham and Women’s Hospital, Harvard Medical School, Boston, Massachusetts, USA

**Keywords:** advisory, economics, fish, methylmercury, nutrition, ocean ecology, poly-chlorinated biphenyls, poly-unsaturated fatty acid, toxicology

## Abstract

Background: Diverse perspectives have influenced fish consumption choices.

Objectives: We summarized the issue of fish consumption choice from toxicological, nutritional, ecological, and economic points of view; identified areas of overlap and disagreement among these viewpoints; and reviewed effects of previous fish consumption advisories.

Methods: We reviewed published scientific literature, public health guidelines, and advisories related to fish consumption, focusing on advisories targeted at U.S. populations. However, our conclusions apply to groups having similar fish consumption patterns.

Discussion: There are many possible combinations of matters related to fish consumption, but few, if any, fish consumption patterns optimize all domains. Fish provides a rich source of protein and other nutrients, but because of contamination by methylmercury and other toxicants, higher fish intake often leads to greater toxicant exposure. Furthermore, stocks of wild fish are not adequate to meet the nutrient demands of the growing world population, and fish consumption choices also have a broad economic impact on the fishing industry. Most guidance does not account for ecological and economic impacts of different fish consumption choices.

Conclusion: Despite the relative lack of information integrating the health, ecological, and economic impacts of different fish choices, clear and simple guidance is necessary to effect desired changes. Thus, more comprehensive advice can be developed to describe the multiple impacts of fish consumption. In addition, policy and fishery management inter-ventions will be necessary to ensure long-term availability of fish as an important source of human nutrition.

The public receives fish consumption advice from a variety of perspectives, including toxi-cant, nutritional, ecological, and economic viewpoints. For example, U.S. federal and state agencies that are concerned about exposure to toxi-cants in fish, such as methyl-mercury (MeHg) and polychlorinated biphenyls (PCBs), have issued fish consumption advisories recom-mending that at-risk groups limit consumption of fish [U.S. Environmental Protection Agency (EPA) 2004]. However, national organizations of physicians and nutritionists encourage fish consumption for the entire population as a way to increase dietary intake of the n-3 (omega-3) long-chain polyunsaturated fatty acids (LCPUFAs) that may prevent cardio-vascular disease and improve neuro-logical develop-ment (Kris-Etherton et al. 2002; Kris-Etherton and Innis 2007; Lee et al. 2009). Also, environmental groups have recommended that consumers avoid certain fish on the basis of concerns about species depletion or habitat destruction consequent to farming methods, site of origin, or type of harvesting (Monterey Bay Aquarium 2011). Whether, how much, and what type of fish a person eats are also influenced by economic and market considera-tions (e.g., cost and availability) as well as by taste, cultural tradition, recreational habits, and access to alternative foods.

Thus, the consumer who wants to know “which fish should I eat?” is likely to encounter contradictory advice, especially because much of the available information considers a single perspective, such as maximizing health or minimizing ecological harms. For example, because farm-raised salmon is high in n-3 fatty acids and very low in mercury, it is promoted for its nutritional benefits. However, environmental groups consider it a “fish to avoid” because salmon aqua-culture may adversely affect eco-system integrity and wild fish stocks (Monterey Bay Aquarium 2011), and relatively high levels of PCBs have led to concerns about cancer risk (Hites et al. 2004). Furthermore, it may be difficult for consumers to know whether any given fish is “good” to eat because they often do not have access to the facts they need to make fully informed choices, such as the size of the fish or how or where it was caught.

Recent articles as well as detailed scientific reports have simultaneously addressed both the nutritional and toxicological aspects of fish consumption [Food and Agriculture Organization of the United Nations (FAO)/World Health Organization (WHO) 2011; Mahaffey et al. 2011; Nesheim and Yaktine 2007; WHO/United Nations Environment Program 2008]. We have been unable to identify any review that addresses the full scope of relevant perspectives (toxicant, nutritional, ecological, and economic) and that has a primary focus on the complexity of balancing these four perspectives. The goal of this review was to extend the fish consumption discussion beyond the toxicant harm–nutritional benefit dichotomy that, although clearly of public health importance, neglects a number of critical issues regarding fish consumption, including the sustainability of fish as a food source. In doing so, we highlight areas of overlap and disagreement among the perspectives. Our broader perspective may complicate fish consumption choices but has the potential to benefit all points of view. For example, the economic viability of the fishing industry depends on the maintenance of adequate fishing stocks. Similarly, nutritional recom-mendations to increase fish consumption will be feasible only if sufficient fish supplies are available to meet greater demand.

## Methods

A group of collaborating authors with complementary expertise in environmental toxicology, nutritional epidemiology, aquatic ecology, economics, and public health practice together defined the outline and scope of this study. We then reviewed published literature as well as guidance disseminated by special interest and professional organizations. We also reviewed experience with previous advisories in the United States.

We primarily focused on issues relevant to purchasers/consumers of store-bought rather than self-caught fish. Because of regional variability in fish species consumed and their respective profiles, we chose to concentrate on consumption advice and guidelines from the United States, including the federal govern-ment as well as state, tribal, and local governments. However, because modern fish production is largely a multi--national industry, we took a more global perspective on the economic impact of fish. Similarly, fish contaminant toxicities or nutrient benefits are applicable to all populations, although we highlight areas where changes in fish intake might have different impacts, for example, among very low or very high consumers. Given the large scope of this article, we did not attempt a comprehensive review of each topic. Rather, we chose to highlight aspects of each perspective that are particularly likely to create confusion (such as the fact that both nutrients and toxicants in fish may influence the same body systems) or that have attracted the most public attention (such as the widely disseminated pocket cards focused on ecological sustainability) (Monterey Bay Aquarium 2011).

## Results

*Perspectives on fish intake.* Toxicant exposure and health risks. Dietary intake of fish and seafood is the dominant source of human exposure to MeHg, a toxicant that can have serious adverse effects on a number of body systems, especially the nervous and cardio-vascular systems. Mercury is a widespread contaminant found throughout the environ-ment [National Research Council (NRC) 2000]. MeHg, an organic form that is converted from inorganic mercury primarily by micro-organisms in the aquatic environment, is biomagnified in aquatic food webs, so the highest concentrations occur in large and long-lived predatory fish and marine mammals at the top trophic levels (NRC 2000).

Community-wide MeHg poisonings in Japan and Iraq highlighted the tragedy of high-dose MeHg exposure as well as the particular sensitivity of the developing fetus (Bakir et al. 1973; Harada 1995). Offspring who were exposed to MeHg *in utero* were born with serious neuro-logical damage, even if their exposed mothers were virtually unaffected (Harada 1995; Igata 1993). Subsequent epidemiological studies among island populations have found more subtle adverse effects of lower levels of MeHg exposure from habitual fish consumption during pregnancy, which have been extensively reviewed elsewhere (Clarkson and Magos 2006; NRC 2000).

Based on evidence for neuro-developmental toxicity from these birth cohort studies, the U.S. EPA recom-mended a MeHg reference dose (RfD) of 0.1 µg/kg body weight per day (NRC 2000). The RfD is an estimate of a daily oral exposure to the human population (including sensitive subgroups) that is likely to be without an appreciable risk of deleterious effects during a lifetime (Rice et al. 2003). The U.S. EPA also incorporated a 10-fold “uncertainty factor” to allow for differences in susceptibility, distribution, and elimination (Rice et al. 2003). However, recent studies in U.S. populations have found evidence for childhood neuro-develop-mental effects of pre-natal MeHg exposure even below the RfD, as reviewed by Karagas et al. (2012).

In addition to MeHg, many other pollutants can be found in fish, including PCBs and other persistent organic compounds, heavy metals, and “contaminants of emerging concern” such as pharmaceuticals and perfluorinated organic compounds. Many of these compounds have established health effects; for example, PCB exposure has been associated with neuro-development and cancer risk (Knerr and Schrenk 2006; Korrick and Sagiv 2008). However, in contrast to MeHg, fish is typically not the only route of exposure to these other contaminants. Furthermore, because contaminant content often varies regionally, advisories to limit exposure to other pollutants focus on the water source as well as the species of fish (U.S. EPA 2010).

Almost all fish are contaminated, to a greater or lesser degree, with environ-mental pollutants. Therefore, the more fish consumed, on average, the more likely an individual is to be exposed to MeHg and other environmental toxicants. Consumers who eat fish frequently or consume highly contaminated species may exceed exposure thresholds. Data from the National Health and Nutrition Examination Survey (NHANES) suggest that about 5–10% of U.S. women of child-bearing age have blood mercury levels consistent with intake exceeding the RfD (Mahaffey et al. 2004). Although debate is ongoing, older women and men may also be at risk; a somewhat less consistent litera-ture has suggested that MeHg exposure from fish consumption in adulthood may be associated with an increased risk of acute coronary events, cardio-vascular mortality, and neuro-logical symptoms (Karagas et al. 2012; Roman et al. 2011).

Nutrient benefits. Fish is high in protein and low in saturated fats and contains a number of other healthful nutrients such as vitamin D, selenium, and iodine. In particular, fish is the primary dietary source of n-3 LCPUFAs, including docosahexaenoic acid (DHA) and eicosa-pentaenoic acid. Because n-3 fatty acids are essential nutrients and because metabolism of the parent n-3 fatty acids to the more biologi-cally active long-chain versions is insufficient in some populations (Mahaffey et al. 2011), dietary intake from fish or from enriched foods and/or supplements is necessary to obtain adequate levels.

Much of the research examining the possible adverse health effects of suboptimal dietary n-3 LCPUFAs has focused on either develop-mental outcomes associated with peri-natal exposure or cardio-vascular risks among older adults. Other outcomes have been also associated with n-3 LCPUFAs (McManus et al. 2009), but in this study we focused on these two end points because of their parallel susceptibility to both nutrient intake and MeHg exposure.

DHA is a necessary structural component of the brain and eye, and the pre- and post-natal periods are likely a critical period for incorporation into these neural tissues (Innis 2000). These anatomic observations have been supported by findings from animal and some human studies (Anderson GJ et al. 2005; Anderson JW et al. 1999; Brion et al. 2011; Innis 2000; Kramer et al. 2008). However, meta-analyses of randomized trials have not found evidence of persistent bene-ficial effects of LCPUFA supplementation of formula milk on the physical, visual, and neuro-develop-mental outcomes of term or pre-term infants (Simmer et al. 2008a, 2008b). Limited evidence from randomized trials of fish oil supplements in pregnancy supports a cognitive bene-fit for offspring (Dunstan et al. 2008), although other trials found no bene-ficial effects (Helland et al. 2008; Makrides et al. 2010).

Cohort studies in the Faroe Islands, Seychelle Islands, and New Zealand focused on associations between pre-natal mercury levels and child develop-ment (NRC 2000). More recent cohort studies that have examined the relation-ship of pre-natal fish consumption with these outcomes have been generally consistent in showing either no adverse effects or improved neuro-develop-ment among children whose mothers ate more fish in pregnancy (Budtz-Jørgensen et al. 2007; Gale et al. 2008; Hibbeln et al. 2007; Lederman et al. 2008; Oken et al. 2005, 2008a, 2008b). Thus, available data suggest that maternal intake of fish and perhaps, although less convincingly, n-3 LCPUFA supplements has modest beneficial effects on neuro-develop-mental and cognitive outcomes of offspring. However, the conclusions that can be based upon these data are limited by a number of factors, including the potential for other neuro--protective nutrients in seafood (e.g., selenium and iodine) to be relevant, and the extent to which confounding (e.g., seafood intake as a marker of healthy lifestyle) explains observed results.

A larger and more consistent body of evidence supports a beneficial role of n-3 LCPUFAs in preventing cardio-vascular disease. Observational studies have found that higher habitual fish intake and higher blood levels of n-3 LCPUFAs are associated with lower risks for congestive heart failure, myocardial infarction, sudden cardiac death, and stroke, as reviewed by Mozaffarian and Rimm (2006).

Although empirical evidence is lacking for the optimal amount of daily n-3 LCPUFAs intake, consensus guidelines recommend DHA intake of about 100–300 mg/day in pregnancy (Akabas and Deckelbaum 2006; Koletzko et al. 2007) and 250–1,800 mg/day for primary prevention of cardio-vascular disease (Kris-Etherton et al. 2002; Mozaffarian and Rimm 2006). Most people consume much less; for example, among U.S. adults in the 1999–2002 NHANES, mean combined intake of DHA plus eicosa-pentaenoic acid was 103 mg/day (Nesheim and Yaktine 2007). Nutritionists and these consensus guidelines have encouraged people to increase their intake of fish to achieve recommended n-3 LCPUFA intake. However, different fish types provide very different amounts of n-3 LCPUFAs. For example, weekly consumption of 6 ounces of shrimp, pollock, or salmon provides an average of 35, 100, and 350 mg/day DHA, respectively (U.S. Department of Agriculture 2009).

Integration of health risks and benefits of fish consumption. Confusion regarding which fish are healthful to eat likely resulted from the fact that early studies assessing the health risk of toxicants found in fish (e.g., MeHg, PCBs) did not incorporate the potential health bene-fits of co-occurring nutrients, and vice versa. Several analyses have attempted to calculate the net health effects of different fish types using estimates of both toxi-cant and nutrient influences (Burger and Gochfeld 2005; Cohen et al. 2005; Ginsberg and Toal 2000; Mahaffey et al. 2011; Stern 2007; Stern and Korn 2011; Tsuchiya et al. 2008). Additionally, a few recent studies, including cohorts focused on child neuro-development (Lynch et al. 2010; Oken et al. 2008b) and adult cardio-vascular disease (Mozaffarian et al. 2011), estimated intake or measured levels of both MeHg and n-3 LCPUFAs.

These analyses will contribute to a clearer picture of the interactions of MeHg and n-3 LCPUFAs on health outcomes, which will allow for guidance to the public that minimizes apparently confusing and conflicting messages about the health effects of fish consumption. However, ecological and economic perspectives, which are generally not considered in analyses weighing possible harms and benefits for health, may result in fish consumption advice or choices antagonistic to recommendations based solely upon human health.

Ecological concerns. Although fish consumption may directly influence human health, human influences, including the harvesting of wild or farmed fish, can profoundly affect the health of the oceans. The rapid decline in large migratory fish species such as tuna, swordfish, and shark has been well documented (Baum et al. 2005; Myers and Worm 2005; Pauly et al. 2002; Worm et al. 2009). Abundance of wild fish stocks is expected to decline further in the future with the added stress imposed by climate variability and habitat alteration, particularly for heavily over-fished stocks that are more sensitive to climate variability (Worm and Myers 2004).

**Figure 1:**
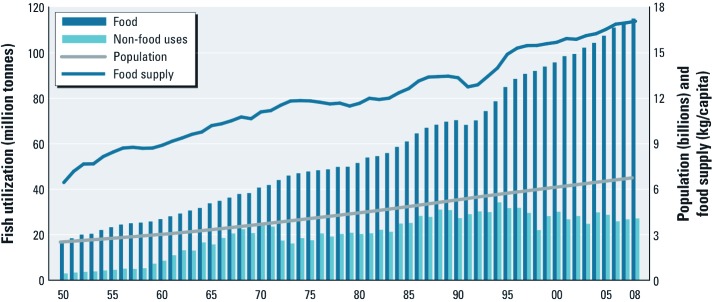
World fish use and supply from 1950 to 2008. Reproduced from FAO (2010b) with permission from the Food and Agriculture Organization of the United Nations.

Globally, the volume of fish production has increased 8-fold since 1950, from about 15 to 120 million tons/year (Figure 1) (FAO 2010b). In part because opportunities for additional harvests of wild fish stocks are limited (i.e., additional harvest could result in species collapse from over-fishing), aqua-culture has grown at a rate of 7–9% per year in the past decade, making it the fastest growing food production industry in the world (FAO 2008). Presently, farmed fish account for 23% of the fish consumed (FAO 2010b). Only one-third of total aqua-culture production is used directly for human consumption, with the remainder used for meal in other farming operations (Rice and Garcia 2011).

Pauly et al. (2002) estimated that global fishing efforts exceeded the maximum sustainable yield by a factor of 3–4. Future needs will likely be even more over-whelming. An estimated 50% increase in fish production is needed by 2050 to meet the basic protein requirements of a growing human population and ensure global food security (Rice and Garcia 2011). If people try not only to meet their protein needs but also to ingest the recom-mended amount of n-3 LCPUFAs from fish, an even greater increase in fish consumption would result.

Creative solutions are needed to resolve the predicament of increasing human demand for fish protein and nutrients amid growing concerns about the global viability of wild fish stocks. Aquaculture has received negative attention because of concerns about the escape of exotic or genetically modified farmed fish species, infection of wild fish stocks with parasites that thrive in farming operations, trophic inefficiencies, enhancement of fish contaminant content, and farm-induced organic enrichment of coastal eco-systems that disrupts their natural functioning (Carniero 2011; Greenberg 2010; Hargrave et al. 1997; Hites et al. 2004; Vanhonacker et al. 2011). However, fish-farming operations can be improved with proper situation of cage sites in estuaries with the appropriate physical conditions (flushing rates and oxygen status) and a focus on lower-trophic-level species such as catfish and tilapia to maximize productivity (Rice and Garcia 2011). Because markets, trade, and consumption patterns strongly influence the activities of the aquaculture community, consumer awareness and demand for sustainable farming practices and quality products can help shape this industry in the future (Khan 2010; Subasinghe et al. 2009).

Economic perspectives on fish consumption. Fisheries are big business on a national and global scale. Indeed, this industry, as well as related industries such as restaurants and grocery purveyors, are key determinants of the amount, type, and form of fish that people consume by affecting the cost, availability, and desirability of different fish.

The United States is one of the world’s largest exporters of seafood products and the world’s second largest seafood importer (Brooks et al. 2009). Over the past half-century, total global production of seafood products has continued to increase, reaching 142 million tons in 2008; the total value of global aquaculture production was estimated at $98.4 billion in 2008 (FAO 2010a). It is obvious from these figures that fish consumption choices have a broad economic impact on the fishing industry, and therefore it is not surprising that this industry seeks to influence the public debate surrounding the harms and benefits of fish intake.

One example of this type of industy influence is canned tuna, a longtime staple in the American diet and the second most commonly consumed type of seafood in the United States; it is also the top dietary contributor to MeHg intake (Groth 2010). There has been an on-going debate regarding whether canned albacore tuna should be listed as a high-mercury fish. The U.S. Food and Drug Administration (FDA) did not include tuna among the high-mercury fish named in its 2001 mercury advisory. Subsequently, a non-profit organization filed a Freedom of Information Act request to access the documents related to the advisory (Nestle 2006). These documents revealed that the FDA had planned to list albacore tuna among the high-mercury fish but dropped the warning after meeting with representatives of the fishing industry. This example illustrates how the interests guiding a fish advisory are not necessarily limited to public health concerns. In fact, the FDA’s regulatory mission is to balance consumers’ health risks against industry interests, such as maintaining demand for popular fish. These issues are not unique to the United States. Although the bluefin tuna used in sushi is high in mercury and ecologically fragile, a recently proposed international ban on bluefin fishing failed after it was vetoed by a number of countries, including Libya, Cyprus, Malta, Spain, France, and Italy, all of which border the Mediterranean and have a stake in the trade of this highly profitable fish (Abend 2010).

As another example, Chilean sea bass has emerged as one of the most popular and profitable fish in U.S. restaurants (Cascorbi 2006). This fish was formerly inaccessible because of its habitat deep in the seas surrounding the Antarctic shelf, as well as being somewhat unappealing when labeled with its official name, the Patagonian toothfish. The rapid expansion of the toothfish fishery in the early 1990s has been linked to the introduction of new fishing techniques as well as aggressive marketing, especially by restaurants, where > 40% of sales occur (Cascorbi 2006). U.S. imports of toothfish, which account for almost half of the worldwide catch, doubled in quantity and tripled in value from 1998 to 2003, from $10 million to > $30 million (Cascorbi 2006). This expansion occurred despite the fact that toothfish are high in mercury (Environmental Defense Fund 2008), vulnerable to overfishing, and caught with methods that result in substantial damage to the seafloor and bycatch of marine birds (Cascorbi 2006).

*Fish consumption advisories and advice.* U.S. Federal governmental fish consumption advisories and their effects. After an NRC review of the health effects of MeHg (NRC 2000), federal and state agencies established fish consumption guidelines based on species-specific mercury levels. In January 2001, the FDA disseminated a consumer advisory on mercury in fish directed at groups considered to be at highest risk: women who might become pregnant, women who are pregnant, nursing mothers, and young children (FDA 2001). The advisory recommended avoiding the four most contaminated fish species (shark, swordfish, king mackerel, and tilefish) and limiting overall consumption of fish and shellfish to ≤ 12 ounces/week (FDA 2001). In 2004, the FDA and the U.S. EPA jointly published a revised advisory that emphasized the nutritional benefits of fish, added a suggested restriction in consumption of canned white (albacore) tuna, and included examples of specific species that are low in MeHg (U.S. EPA 2004). These changes were welcome because many consumers may have been more aware of the content and effect of harmful substances in fish than of the nutrients (Bloomingdale et al. 2010; Verbeke et al. 2005).

Several investigators have taken advantage of existing data sets to estimate effects of the U.S. federal government mercury advisories on fish consumption. In a cohort study of well-educated pregnant women in Massachusetts that straddled dissemination of the FDA advisory (FDA 2001), women reported consuming less dark meat fish, canned tuna, and white meat fish after publication of the advisory (Oken et al. 2003). Using a panel of nearly 15,000 U.S. households, Shimshack and Ward (2010) studied fish purchases from 2000 through 2002, finding that households with pregnant women or young children reduced both their mercury and n-3 LCPUFA intakes after the 2001 advisory. The n-3 LCPUFA decline occurred everywhere along the distribution of intakes, including among those with the lowest intake. Results were driven by a broad-based decline in consumption of all fish. On average, consumers, even those with a college education, did not differentially avoid high-mercury fish, nor did they substitute away from high-mercury species into low-mercury, high-omega-3 species. However, less educated households showed no advisory-induced reduction in mercury (Shimshack and Ward 2010).

In contrast, NHANES data indicated that blood mercury decreased from 1999 through 2004, without a concomitant decrease in fish consumption (Mahaffey et al. 2009). Although the cause for this decrease remains unclear, the authors speculated that the findings suggested a more discerning series of choices in type of fish eaten rather than an overall reduction in fish consumption (Mahaffey et al. 2009)

Most recently, an analysis using pooled nationally representative 2001 and 2006 food safety surveys indicated an increase in U.S. consumers’ awareness of mercury as a problem in fish (69% in 2001 to 80% in 2006), especially among parents of young children (Lando and Zhang 2011). However, women of child-bearing age were less aware and knowledgeable about this information than other women.

U.S. local government fish consumption advisories and their effects. Individual U.S. states and tribes collect data and issue advisories on mercury in fish caught from local bodies of water. Some states and localities provide advice for commercial fish consumption as well (U.S. EPA 2010). Their recommendations may include information on species that are of particular relevance to the local population but not necessarily included in nation-wide U.S. advisories. Advisories differ from state to state based on a number of variables. For example, most advisories target children, pregnant women, and women of child-bearing age, and a few states also provide advice for the general population (Scherer et al. 2008). Although most advisories are based on the U.S. EPA’s RfD for MeHg established in 2000 (NRC 2000), a few are based on the FDA action level established in 1979, which is approximately four times higher (Tollefson and Cordle 1986). A few states (e.g., Alaska) have derived their own health assessments and used these in formulating advice.

Approximately 80% of U.S. fishing advisories are, at least in part, related to mercury contamination. The most recent data indicated that across all 50 states, as of 2010, there were ≥ 4,500 fish consumption advisories (i.e., advice to limit or avoid consuming fish from a given water body because of contaminant risk) (U.S. EPA 2010). These advisories cover 4 of every 10 river miles, almost 79% of contiguous coastal waters, and 40% of all fresh-water surface area in the United States, not including the Great Lakes, 100% of which are under advisories. In contrast, in 2010 only 2% of the nation’s river miles and 9% of the nation’s lake acres had safe-eating guidelines in effect (i.e., an indication that fish from the body of water was safe for consumption) (U.S. EPA 2010).

Awareness of regional fish consumption advisories in the United States is generally low, ranging from 8% to 32% (Anderson et al. 2004; Gliori et al. 2006; Knobeloch et al. 2005). Furthermore, results from several surveys suggest that awareness of regional fish advisories is not more common among higher-risk sub-groups, such as pregnant women, nor does awareness necessarily predict lower mercury levels or less frequent consumption of higher-mercury fish (Burger and Gochfeld 2009; Karouna-Renier et al. 2008; Knobeloch et al. 2005; Silver et al. 2007). Challenges to communicating effectively with high-risk groups have included language barriers, educational and literacy status, income level, cultural differences, and difficulty reaching racial/ethnic minority groups (Imm et al. 2007; Kuntz et al. 2009; Silver et al. 2007). In addition to these challenges, many consumers simply do not want any more information. For example, although most surveyed fishers in the New York Bight did not have accurate knowledge on harms and benefits of fish consumption, well over one-third of them did not feel they needed more information (Burger and Gochfeld 2009).

Other resources. In addition to advice issued by the U.S. federal government and states, not-for-profit and other non-govern-mental organizations also provide information on mercury in fish directly to consumers. In Table 1, we summarize a number of fish consumption recommendations for U.S. populations, by target audience and messages that are conveyed. For example, the Natural Resources Defense Council and the Turtle Island Restoration Network provide online mercury calculators that allow consumers to calculate whether their mercury intake exceeds the U.S. EPA RfD, based on their body weight and combinations and amounts of fish species consumed. In Table 2 we list several web sites that link to valuable sources of information for the public regarding fish consumption. Other groups, such as Physicians for Social Responsibility (2004) and the Environmental Working Group (2012), provide lists of fish species with higher and lower mercury concentrations, along with consumption guidelines.

**Table 1:** Summary of major seafood consumption guidelines or advisories targeted at North American populations.

Reference	Target or vulnerable population	Contaminant exposure	Fatty acid/ nutrient intake	Ecological impact	Economic influences	Web site
FDA/U.S. EPA 2004	Women, children					http://www.fda.gov/Food/ResourcesForYou/Consumers/ucm110591.htm
Monterey Bay Aquarium Seafood Watch 2012	General population					http://www.montereybayaquarium.org/cr/cr_seafoodwatch/sfw_recommendations.aspx?c=ln
Environmental Defense Fund 2008	General population					http://apps.edf.org/page.cfm?tagID=1521
USDA and Department of Health and Human Services 2010	General population, women					http://www.health.gov/dietaryguidelines/2010.asp
Fish4Health.net 2009	Women, children					http://fn.cfs.purdue.edu/fish4health
Blue Ocean Institute 2012	General population					http://www.blueocean.org/seafood/seafood-guide
Kidsafe 2012	Children					http://www.kidsafeseafood.org/
Fishwise 2012	General population, retailers					http://www.fishwise.org/science/purchasing-tools/
Washington State Department of Health 2011	General population, women, children					http://www.doh.wa.gov/ehp/oehas/fish/default.htm
State of Connecticut Department of Public Health 2012	General population, women, children, avid fish eaters, fishers					http://www.ct.gov/dph/cwp/view.asp?a=3140&Q=387460
Natural Resources Defense Council 2009	General population					http://www.nrdc.org/oceans/seafoodguide/default.asp
Turtle Island Restoration Network 2012	General population					http://www.gotmercury.org
Food and Water Watch 2011	General population					http://www.foodandwaterwatch.org/fish/seafood/guide
Mercury Policy Project 2010	General population, women, children					http://www.mercuryfactsandfish.org/
National Geographic 2012	General population					http://ocean.nationalgeographic.com/ocean/take-action/impact-of-seafood/#/seafood-decision-guide/
Star Chefs 2004	Chefs					http://starchefs.com/features/food_debates/html/sustainable_seafood.shtml
Greenpeace International 2012	General population, retailers					http://www.greenpeace.org/international/seafood/
National Oceanic and Atmospheric Administration 2012	General population					http://www.nmfs.noaa.gov/fishwatch
Shedd Aquarium 2012	General population					http://www.sheddaquarium.org/3163.html
Health Canada 2007	General population, women, children					http://www.hc-sc.gc.ca/fn-an/pubs/mercur/merc_fish_poisson-eng.php
Institute of Medicine 2006	General population, women, children, adults at risk for cardiovascular disease, avid fish eaters					http://www.iom.edu/Reports/2006/Seafood-Choices-Balancing-Benefits-and-Risks.aspx
Light shading indicates that the topic is addressed in part; dark shading indicates that the topic is addressed in detail.						

**Table 2:** Selected web sites with links to seafood guides.

Sponsor	Web site
Seafood Choices Alliance	http://www.seafoodchoices.org/resources/links.php#linksseafoodcards
Stonybrook University	http://www.stonybrook.edu/commcms/gelfond/fish/advice.html
University of Rhode Island Sustainable Seafood Initiative	http://www.seagrant.gso.uri.edu/sustainable_seafood/guides.html#list

Other guides incorporate information advocating ocean conservation and warning of the environmental hazards associated with certain types of seafood consumption. Popular guides such as the Monterey Bay Aquarium Seafood Watch (Monterey Bay Aquarium 2011) combine information about the sustainability of fisheries and catch methods with information on contaminant burdens and nutrients in different species.

Challenges for fish consumption choice. Considerable uncertainty exists regarding the actual toxicological, nutritional, ecological, and/or environmental harms and benefits of consuming any given fish. Among the hundreds of species of fish available for consumption, characteristics are highly variable. Even within species, nutritional, contaminant, and ecological attributes can vary widely depending on the size or variant or where the fish is harvested or farmed. For example, shrimp can be rated as an ecological “best choice,” “good alternative,” or “avoid” depending on its origin (Monterey Bay Aquarium 2011). Similarly, tilefish caught in the Gulf of Mexico is very high in MeHg, whereas tilefish from the Atlantic Ocean is low in MeHg (Sunderland 2007).

Furthermore, there is variation in susceptibility to the benefits or harms of fish consump-tion among individuals by age and other characteristics. Also, the net health effect of a change in intake for each individual (or popu-la-tion) depends on baseline intake: If intake is low, the net harm of a further reduction is likely to be greater than if intake is high (Hammitt 2004).

Incomplete information may result in expert advice that is incorrect or mis-leading. For example, most U.S. commercial fish consumption advisories to limit MeHg exposure are based on mean or median mercury concentrations measured in fish samples collected by the FDA. However, these reference data may be based on a small number of fish and are often not up-to-date, and mercury concentrations may vary widely even within the same species. For example, some samples of high-mercury species such as swordfish may have non-detectable levels of mercury, whereas lower-risk species such as halibut may have levels > 1 ppm (FDA 2011). In a recent study of different eco-labels for farmed fish, Volpe et al. (2011) found no evidence that these certified products are actually environmentally preferable, in part because many of the standards applied in the different labels ignored major environmental impacts.

Once advice is issued, consumers may not respond in ways that result in better outcomes. Economic wisdom holds that improved information enhances welfare because consumers refine and adapt their consumption in response to new information. However, it is not clear whether welfare actually increased after the FDA’s seafood consumption advisories (Blanchemanche et al. 2010; Shimshack and Ward 2010). First, rather than substituting higher-mercury fish for lower-mercury fish to reduce exposure while still obtaining benefits provided from fish, many consumers simply reduced their over-all fish intake, which also resulted in a decreased intake of nutrients obtained from fish. Second, although the FDA’s advice targeted pregnant and breast-feeding women, even non-targeted adults reduced their fish consumption (Shimshack and Ward 2010; Shimshack et al. 2007). These consumers may have incurred a welfare loss because their reduction in fish intake led to a reduced intake of n-3 LCPUFAs and therefore increased cardio-vascular risk (Mozaffarian and Rimm 2006), possibly out-weighing the gains from decreased fish intake (e.g., from decreased MeHg exposure).

Why would people make choices that may actually worsen, rather than improve, their health? Balancing risks is notoriously difficult. When individuals make judgments under uncertainty, they tend to use a limited number of cognitive processes. These processes are efficient but can sometimes lead to errors or biases (Kahneman 2003). People often over-estimate some risks (e.g., the risk of harm from MeHg exposure), whereas they under-estimate others (e.g., the risk of harm from sub-optimal nutrition) (Slovic et al. 2000). They tend to focus on worst-case scenarios (Viscusi 1997). Many consumers are better aware of the content and effects of harmful substances than of nutrients in fish (Verbeke et al. 2005).

Given these uncertainties, consumers are likely to employ a bounded rationality approach to make consumption choices (McFadden 2001). That is, they recognize that the gathering and processing of information comes at a cost in terms of time and cognitive burden. Instead of striving for more information to update their beliefs about the relevant health risks, they eventually adopt simpler heuristics to make consumption choices (Gigerenzer and Goldstein 1996). The fact that consumers not targeted by the FDA’s 2001 mercury advisory (FDA 2001) reduced their fish consumption (even of fish lower in mercury) simply to rule out a food risk is consistent with the bounded rationality assumption.

Messages that are simple or that are targeted at well-known fish species are more likely to be effective (Verger et al. 2007). In focus groups, participants preferred simple messages; however, they did not always respond appropriately (Nesheim and Yaktine 2007). For example, almost all participants reported that they would avoid species desig-nated “do not eat” regardless of whether they were in the targeted audience. Also, responses vary depending on whether “risks” or “bene-fits” are listed first (Knuth et al. 2003; Verbeke et al. 2008).

## Discussion

The possible combinations of matters related to fish consumption—including toxico-logi-cal, nutritional, ecological, and economic—are many, but few, if any, fish consumption patterns optimize all four of these areas. In Table 3 we summarize these viewpoints and the challenges they present to comprehensive advice.

**Table 3:** Challenges to developing comprehensive fish consumption advice.

Viewpoint and challenges	Examples
Toxicological hazards: fish contaminants (e.g., MeHg, PCBs, pesticides)		
Multiple co-occurring contaminants		Synergistic adverse effects on neurodevelopment with joint MeHg and PCB exposure
Advisories for single contaminant		Farmed salmon low in MeHg but can be high in PCBs
Toxicant levels vary within and across species		Tilefish MeHg: high in Gulf of Mexico but low in Atlantic
Variable susceptibility to toxicities		Prenatal exposure: increased susceptibility to MeHg neurotoxicity
Confounding by nutritional benefits		Underestimation of hazard from MeHg if confounded by n‑3 LCPUFAs
Nutritional benefits: fish nutrients (e.g., n‑3 LCPUFAs, vitamin D, iodine, selenium)		
Multiple co-occurring nutrients		For neurodevelopment, fish intake more consistently beneficial than n‑3 LCPUFA supplementsa
Nutrient levels vary within and across species		Health benefits associated with high n‑3 LCPUFA fisha
Confounding by contaminant risk		Underestimation of benefit from n‑3 LCPUFAs if confounded by MeHg
Increased fish intake (for most populations) recommended by nutrition guidelines		Available fish insufficient to meet demand even without greater intake
Environmental sustainability: overfishing, habitat destruction, aquaculture		
Modern harvesting can deplete fish stocks, other aquatic wildlife, and habitats		Fishing trawls plough the seafloor, removing most, if not all, aquatic life and structures in their path
Wild fish stocks insufficient to meet projected global demand		Approximately 90% of large predatory fish stocks (e.g., bluefin tuna, Atlantic salmon) are already depleted
Aquaculture can adversely affect wild fish and ecosystems; contaminants		Farmed salmon can escape and outcompete wild fish and may have higher PCB levels
Economic influences: consumer choice, industry stakeholders, fisheries management		
Economic assumption of improved consumer welfare with more information not necessarily true for fish choice		U.S. federal advisories led to overall decrease in fish and n‑3 LCPUFA consumption, not just among targeted species and consumers
Cost and availability influence choice		Choose less expensive but less nutritious food (e.g., high-fat meat)
Fishing is a huge global industry influencing consumers, fisheries management, and regulatory structure		The fishing industry lobbied successfully to keep albacore tuna out of the 2001 U.S. FDA advisory
Industry’s economic interests often in conflict with toxicant, nutritional, and environmental interests		Profitability encourages promotion of bluefin tuna production despite high MeHg, high trophic level, and species endangerment
aMultiple fish nutrients may be important to observed beneficial associations of fish intake with health.		

Individual and market economics can influence seafood consumption decisions in ways that may be largely independent of specific toxicant hazards, nutrient benefits, or eco-system effects. In addition, availability, taste preferences, cultural traditions, and cost affect consumers’ fish intake (Verbeke and Vackier 2005). Ecological and economic impacts of fish choice are perhaps the least “visible” to consumers and therefore the most difficult to incorporate into decision making (Verbeke et al. 2007). Furthermore, when consumers choose not to eat fish, regardless of the reason, the foods eaten instead (e.g., red meat) also may have variable health, ecological, and economic impacts.

The future of fish advisories is a matter of ongoing debate and presents a number of alternative options. Agencies may recommend that populations of highest concern refrain from eating fish with high concentrations of MeHg, similar to the FDA advisory (FDA 2001) and many state advisories. But past experience has shown that this approach excludes many “low-risk” populations that may in fact suffer harm from MeHg toxicity, and also is likely to reduce fish intake indiscriminately, worsening nutrition. An alternative approach is to suggest that people should eat fish, without parsing out the contaminant or ecological harms of different fish types. For example, the 2010 *Dietary Guidelines for Americans* (USDA 2010) encourage everyone, including pregnant and breast-feeding women, to eat seafood at least twice a week. However, this advice might expose a subset of the population to risk of substantial harm from increased MeHg intake and is likely unsustainable given the projected inadequacy of fish stocks to support population growth, even at current consumption levels.

More comprehensive advice that describes both the potential hazards and benefits of fish consumption can be developed. However, such an approach is constrained by a relative lack of information integrating not only health risks and benefits but also ecological and economic impacts. Furthermore, experience to date suggests that effective communication of multiple competing risks is difficult at best and, at worst, may encumber consumers with irreconcilable risk–risk trade-offs. Additionally, although consumer demand for healthful, sustainably harvested or farmed fish can help shape fishing industry practices, it is unlikely that consumers alone can substantially influence these practices. Policy and fishery management inter-ventions will be necessary to ensure long-term availability of fish as an important source of human nutrition.

## Conclusion

On an individual level, decisions regarding which fish to eat—and whether to change fish consumption habits—may vary widely across consumers. We have not yet met the challenge of providing consumers with accessible information that includes nutritional, contaminant, ecological, and economic trade-offs associated with fish consumption choices, including guidance to consumers who vary by baseline intake, life stage, and reliance on fish intake because of subsistence needs or cultural traditions.

Based on evidence we present here, fish consumption advice addressed to the general public should be clear and simple to have an impact. We suggest developing a list of fish to eat, and those to minimize or avoid, that considers these multiple perspectives and not solely the health effects of contaminants and nutrients. This list should include links to more detailed resources that can be used by those wanting more information about individual fish types or wishing to optimize one or more parameters. The simple message needs to be provided on a national level but with the cooperation of local and regional partners (e.g., states and non-governmental organizations). Thus, adjustments could be made on a regional level if necessary, as long as the framework can be followed. As further information becomes available, the list of beneficial choices, as well as choices to avoid, could be improved upon. Although simplicity of messaging is paramount, the under-lying paradigm addressing the challenges presented in Table 3 would not be simple. Yet with transparency, an approach on a national level could be developed that provides clear choices protecting public and global health.

Meanwhile, we should continue to urge international organizations, governments, and agencies to promote remediation and, where possible, elimination of sources of fish contamination and to establish policies that promote environmentally responsible and economically viable fishing practices so fish can remain a part of a healthy human diet for future generations.
